# Real-Time Classification of Anxiety in Virtual Reality Therapy Using Biosensors and a Convolutional Neural Network

**DOI:** 10.3390/bios14030131

**Published:** 2024-03-03

**Authors:** Deniz Mevlevioğlu, Sabin Tabirca, David Murphy

**Affiliations:** 1 School of Computer Science and Information Technology, University College Cork, T12 K8AF Cork, Ireland; tabirca@cs.ucc.ie (S.T.); d.murphy@ucc.ie (D.M.); 2 Faculty of Mathematics and Informatics, Transylvania University of Brasov, 500036 Brasov, Romania

**Keywords:** VRET, biosensors, human–computer interaction, machine learning, affective computing, VR, EDA, PPG, EEG

## Abstract

Virtual Reality Exposure Therapy is a method of cognitive behavioural therapy that aids in the treatment of anxiety disorders by making therapy practical and cost-efficient. It also allows for the seamless tailoring of the therapy by using objective, continuous feedback. This feedback can be obtained using biosensors to collect physiological information such as heart rate, electrodermal activity and frontal brain activity. As part of developing our objective feedback framework, we developed a Virtual Reality adaptation of the well-established emotional Stroop Colour–Word Task. We used this adaptation to differentiate three distinct levels of anxiety: no anxiety, mild anxiety and severe anxiety. We tested our environment on twenty-nine participants between the ages of eighteen and sixty-five. After analysing and validating this environment, we used it to create a dataset for further machine-learning classification of the assigned anxiety levels. To apply this information in real-time, all of our information was processed within Virtual Reality. Our Convolutional Neural Network was able to differentiate the anxiety levels with a 75% accuracy using leave-one-out cross-validation. This shows that our system can accurately differentiate between different anxiety levels.

## 1. Introduction

Anxiety disorder is an umbrella term for mental health conditions involving excessive and disproportionate levels of fear or anxiety. Anxiety, although sometimes helpful in responding to dangerous situations, can be debilitating when excessive. There are many different types of anxiety disorders, ranging from generalised anxiety disorder to social anxiety disorder, from specific phobias to selective mutism [1]. The treatment for anxiety disorders is in response to the particular type of anxiety disorder. The most common treatments would include psychotherapy methods such as cognitive behavioural therapy (CBT) and medication such as selective serotonin reuptake inhibitors (SSRIs) or serotonin–norepinephrine reuptake inhibitors (SNRIs) [2].

Physical symptoms of anxiety disorders can include heart palpitations, shortness of breath and chest pain. Other symptoms can include restlessness, irritability and inability to sleep or concentrate. They significantly impair the patient’s life, making it difficult to carry out daily tasks or work. The aetiology of anxiety disorders constitutes a complex interaction of psychosocial factors such as childhood trauma, stress and genetic predisposition [3].

Anxiety disorders are one of the most prevalent mental health conditions [3]. Therefore, screening, diagnosis and treatment of anxiety disorders are crucial to the well-being of society. There is a growing body of evidence that points to the efficacy of Virtual Reality Exposure Therapy (VRET) for the treatment of anxiety disorders, particularly social anxiety disorder and specific phobias.

One recent control study by Anderson et al. [4] investigating the effectiveness of VRET in treating social anxiety disorder compared to in vivo treatments found no significant differences in any outcome measure and no difference in partial or full remission rates. This has been maintained over a one-year follow-up.

Similarly, a study by Bouchard et al. [5] found improvements to all outcome measures compared to waitlist controls in both in vivo exposure therapy and VRET. Furthermore, there were improvements above in vivo in the post-treatment primary outcome and one secondary outcome. This improvement persisted over a six-month period. Psychiatrists reported that using VRET is more practical than using in vivo [5].

Meta-analyses show no significant improvement in therapy outcomes for VRET over in vivo [6,7]. However, they show an improvement over wait-list and placebo controls [6].

Although the treatment methods are generally comparable to in vivo treatments, no evidence points to improved outcomes using Virtual Reality (VR). They are nonetheless beneficial due to their cost-efficiency [8] and practicality [5]. An experiment by Garcia-Galacios et al. [9] shows that 81% of students preferred VRET to in vivo treatment. However, based on the meta-analysis by Benbow and Anderson [10], the drop-out rates did not show any significant difference.

Accessibility and the helpfulness of VR led many researchers to seek ways to improve VR therapy. One such way is tailoring the therapy environment to the patient’s needs. To do this, however, continuous feedback from the patient is required. Self-report feedback can be unreliable and challenging to obtain during a VR session. Interruptions to exposure to fill in questionnaires can take a toll on the immersiveness of the experience and increase the likelihood of dropout [10]. Therefore, providing objective and non-intrusive feedback during a VR therapy experience is crucial.

Common approaches to measuring anxiety in VR involve using physiological and biological markers for anxiety [11]. Standard physiological measures include heart rate (HR), electrodermal activity (EDA), also known as Galvanic Skin Response (GSR), electrical brain activity and skin temperature (SKT). Regarding behavioural measures, some popular measures include head movement, muscle movement, eye movement and respiration (RESP) [12]. Numerous studies combine machine learning methods and on-body biological sensors for optimal, accurate and objective feedback on anxiety levels [13,14,15].

Extensive research shows that it is possible to measure and categorise distinct anxiety levels using biosensors [12]. However, not all devices used for measuring physiological signals are suitable for use in VR. The highest priority in VR is to allow for user mobility and comfort so users can explore and experience the environment at their own pace [16]. However, a lot of on-body sensors, such as electrocardiograms (ECG), photoplethysmograms (PPG), and electroencephalograms (EEG), work best when the user is stationary [17]. Due to the noise introduced by movement and the electrical interference of the head-mounted display (HMD), it is important to use strategies such as filtering, multi-modality and normalising to offset the negative effects, and while many preprocessing methods on physiological data ensure the highest quality results, not all are viable for real-time use.

A study by Šalkevicius et al. [14] used signals such as EDA, blood volume pressure (BVP), and SKT to classify anxiety levels within VRET. Using public speaking anxiety as the basis of their exposure and stress stimulus, they achieved an 80% cross-participant accuracy among thirty participants using a support vector machine (SVM).

Similarly, Petrescu et al. [18] used HR and EDA features with a regression model and a rule-based system to extract classes from a regression model. They used height as a stress stimulus. From 7 participants, they were able to achieve 92% accuracy. However, the accuracy might be biased due to the uneven distribution of classes in their data. They report that the accuracy for the mild anxiety class was 81%, and for the high anxiety class, it was 76%, based on a confusion matrix.

Cho et al. [19] took a slightly different approach and combined arithmetic tests with VR environment stimulus to elicit the stress response. Their kernel-based extreme learning machine (K-ELM) algorithm was able to differentiate between their two baseline environments (before and after the stimulus) and three anxiety environments (mild, moderate and severe) with 95% accuracy.

Our work focuses on both the comfort and accuracy of our system to seamlessly and objectively measure the anxiety level of the user, between no anxiety, mild anxiety and severe anxiety.

## 2. Materials and Methods

### 2.1. Participants

Twenty-nine volunteers recruited through a large university campus have participated in the study. Eleven participants were between the ages of eighteen and twenty-four (38%), fifteen participants between the ages of twenty-five and thirty-four (51%), two participants between the ages of forty-five and fifty-five (7%) and one participant older than fifty-five (3%). Eleven participants were female (38%), and eighteen participants were male (62%). There were seven participants who had never used VR (24%), fifteen participants who had used it a couple of times (51%), five participants who had one to two years of experience in VR (17%) and two participants with two to five years of experience (7%). Seven participants stated that they had never experienced motion sickness (24%), fourteen participants had experienced it rarely (48%) and eight participants sometimes (28%). Four participants were diagnosed with colour blindness (14%), and three participants were diagnosed with dyslexia (10%).

### 2.2. Framework Design

**Figure 1:**
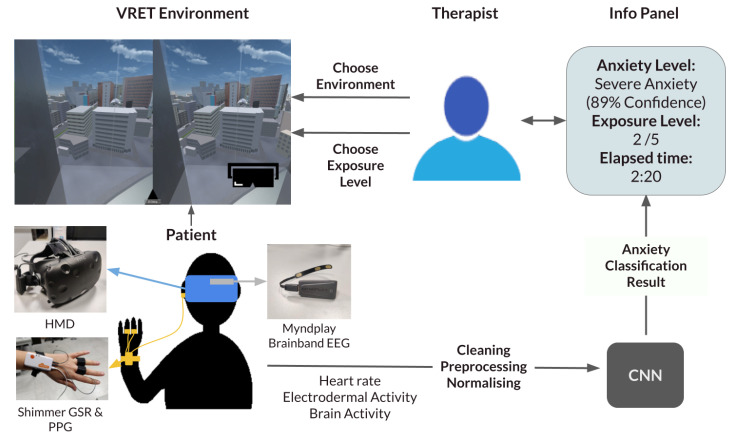
Framework diagram. CNN: Convolutional Neural Network; EEG: electroencephalogram; GSR: Galvanic Skin Response; HMD: head-mounted display; PPG: photoplethysmogram.

Our proposed framework is a generalisable system that inputs different exposure environments and provides a seamless decision-support system for exposure therapy. The therapist can pick between different types of anxiety disorders, choose the exposure environment, and then choose the exposure level. To inform their decision, an information panel continuously updates with crucial information. The information displayed includes the predicted current anxiety of the user, the time of last exposure change and the current exposure level. The therapist can also view the raw data if they please. A diagram summarising the system can be viewed in Figure 1.

Our current experiment informs the classification part of this framework by seamlessly supplying the therapist with the predicted anxiety level in the current exposure.

### 2.3. Experiment Design

This study uses the well-established emotional Stroop Colour–Word Task (eStroop) [20] as a stimulus for anxiety in the VR environment in order to label classification data. EStroop uses words from affective norms for English words (ANEW) [21] to elicit negative arousal from the user. Users are tasked to mark the colour of the words that appear while the words vary in emotion levels. We have created a Virtual Reality version of this task, which we call the emotional Virtual Reality Stroop Task (eVRST). It traditionally has a neutral condition with neutral words and an emotional condition with negative arousal words. We added a third condition to the task, between the neutral and emotional conditions. With this addition, we name the conditions neutral, mildly emotional and severely emotional. The words we used in the experiment can be found in Supplementary Table S1. Using these conditions, we aimed to differentiate between three distinct levels of anxiety. To validate, we used self-report and physiological information. Once the environment was validated statistically, we used machine learning to classify which condition the participants were taking based on physiological information.

### 2.4. Metrics and Devices

We used pulse rate, EDA and electrical brain activity as metrics. Pulse rate was chosen because of the high correlation it shows with anxiety in the literature [22]. It is also convenient and comfortable to capture. Skin conductivity, similarly, was selected due to its strong association with arousal levels [23]. Electrical brain activity is a more complex measure for VR due to HMD electrical inference and difficulty attaining a good signal without preparation and many channels. However, it is an excellent measure of valence, essential for indicating the direction of the arousal, whether the user is anxious or just excited [24]. Heart rate variability (HRV) was not considered for this study because the results were to be used in real-time, and the calculation of HRV over periods below one minute is unreliable [25].

The devices used for measuring the selected physiological information were decided upon based on accuracy, reliability and ease of use. Once again, priority was given to user comfort. Multiple devices were considered for each measure.

Skin conductivity is a fundamental metric when attempting to predict anxiety. Thus, the device selection was weighted towards this direction. The options considered for measuring GSR were the Shimmer GSR+ [26] device and the Empatica E4 [27] device. Shimmer GSR+ contains two GSR finger electrodes. Empatica E4, on the other hand, is a wristband that can be attached around the wrist, making it much more comfortable and easier to attach. Our preliminary tests aligned with the literature on higher accuracy of signals for the Shimmer GSR+ device. Due to the importance of this signal, the Shimmer GSR+ device was favoured over the Empatica E4 wristband.

For heart rate, the technologies considered were ECG and PPG. Based on prior research, ECG is more reliable and accurate in collecting heart rate information. However, they require multiple electrodes to be attached to the body for an optimal signal. Consecutively, we chose to use PPG in our research due to its compactness and comfort. PPG might produce less accurate results, but it is consistent and valuable. When choosing a device for the PPG, two devices were considered: Shimmer GSR+ and Empatica E4. Empatica E4 provided more comfort and comparable accuracy in heart rate measurements. However, as Shimmer GSR+ provided better results for measuring skin conductance, it was deemed optimal to use this device. As Shimmer GSR+ was already being used for skin conductance, also using it to capture PPG ensured our solution was more lightweight and compact. Shimmer GSR+ uses optical pulse sensors for measuring PPG. These can be attached either on the earlobe or around the finger. Based on our preliminary testing, we found that the earlobe lead was more reliable.

When it came to electrical brain activity, an EEG was used. Picking the right tool for this task was challenging due to the availability of varying professional and consumer-grade devices. The first decision was how many channels were appropriate to use. More channels provide higher-quality signals but also make the system more cumbersome. We decided to use a single channel to simplify the system. When it came to using dry nodes or wet nodes for the EEG, similarly, we chose the most comfortable option over higher signal quality and decided on dry nodes. Based on extensive research of the literature and available devices, we decided that the MyndPlay MyndBand [28] was the best fit for us. This device produces valuable results using a single dry node and a signal channel. MyndPlay MyndBand features three sensors, a headband and a Bluetooth unit. We removed the headband and attached the Bluetooth unit and sensors directly to the HTC Vive to make it more comfortable for the users. This made it easier to use and avoid problems such as the headband slipping with the HMD movement.

Participants were asked to complete one questionnaire before and one after the experiment. The first questionnaire included demographic questions and a short version of the State-Trait Anxiety Inventory (STAI-Y1). STAI-Y1 is a standard metric for measuring anxiety levels. It was first developed by Spielberger et al. [29], and we used the version modified by Morteau and Bekker [30] to be shorter.

The second questionnaire asked the users to rate each condition in the experiment between no anxiety, mild anxiety and severe anxiety options. They also were asked for subjective evaluation of the experience based on several 5-point Likert-style questions such as “I found the equipment used during the experiment cumbersome”.

During the experiment, subjective units of distress scale (SUDS) was used to rate each condition after completion. This scale ranks the current distress of the user from 0 to 100, 0 being no stress and 100 being the highest stress imaginable [31].

HTC Vive HMD, modified to include the Myndplay MyndBand, was used to display the VR experience. A desktop computer with a GeForce GFX Titan X graphics card and an Intel i7-5820k processor was used to run the experiment. The audio was delivered through the HTC Vive earphones.

### 2.5. The Virtual Reality Environment

**Figure 2:**
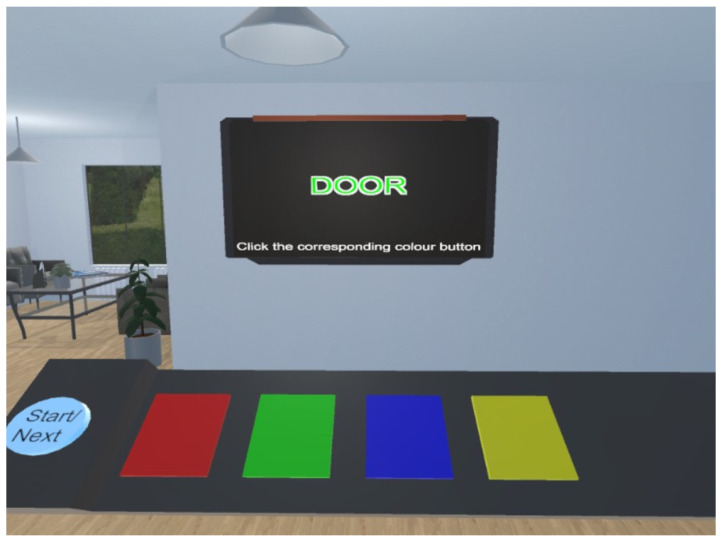
The Virtual Reality waiting room environment used for elicitating of anxiety.

The environment and the task were modelled using the Unity 3D engine [32]. The environment was modelled after a standard clinic waiting room to improve familiarity and comfort, including couches, plants, posters and screens. We used a television screen to display Stroop prompts and provided a visual platform for users to control by moving the controllers (Figure 2). Visual, auditory and haptic feedback are provided upon selecting a button in the form of a yellow button highlight, a clicking sound effect and a short vibration.

The users were provided as much time as they wanted to get accustomed to the VR environment. After this time, they go through a tutorial to learn the task. Audio instructions artificially generated by Murf AI [33] are provided, as well as text on the screens for improved accessibility.

The task itself is designed to be accessible and intuitive. The user has two seconds to answer each prompt. We did not implement time pressure as the experiment progressed to ensure we measured the anxiety produced by the words, not task difficulty.

Each condition has randomly ordered words selected from each list of words: neutral, mildly emotional and severely emotional. Extraneous variables in the experiment are controlled, with only the meanings of the words as the independent variable.

The user fills out their anxiety on the SUDS after each condition, including the tutorial. To do so, they use a VR slider.

An additional scene is used as a fallback should the user get too anxious and decide to exit the experiment early. They do so by clicking a dedicated button on the platform, bringing them to a quiet forested area where they can relax until the experimenter removes the equipment.

### 2.6. Procedure

**Figure 3:**
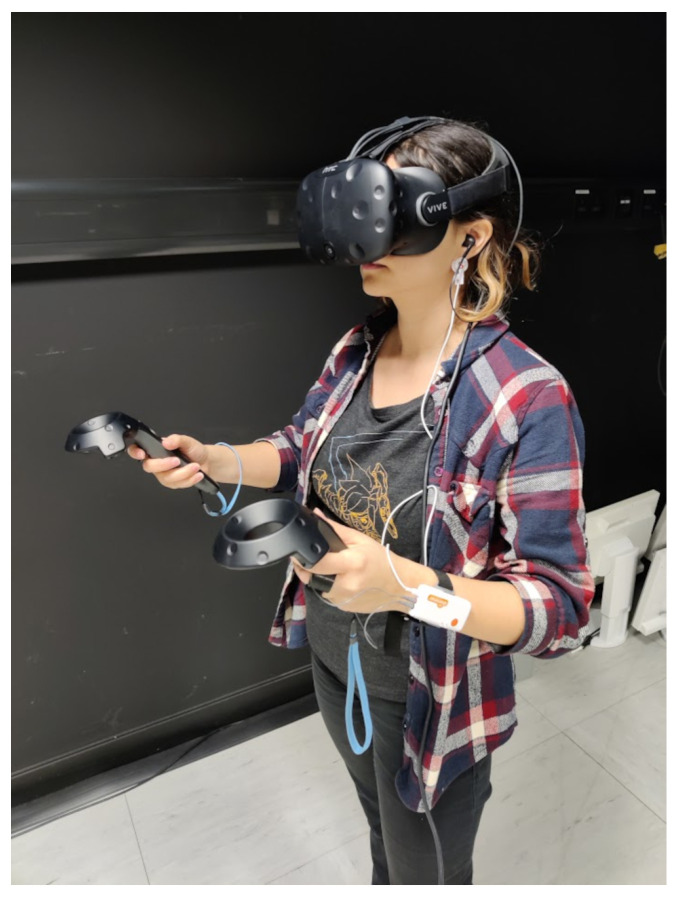
A user with the equipment attached.

Participants read the information sheet that outlined the experiment, data protection, ethics statement and their rights. If the participants were satisfied, they signed the consent sheet. The consent sheet also has their participant number. Once they were ready to start, they were asked to fill in the first questionnaire. Participants were assigned a unique ID to match their second questionnaire and physiological information. This ID is separate from their participant number but is linked to a document. We used two sets of identifiers to allow participants to withdraw within fourteen days, after which we deleted the document that links their information to their consent form. When the participants completed the questionnaire, they were instructed to move to the play area, marked by an “X” on the floor of the VR lab. The experimenter put the HMD on their head, Shimmer GSR+ on their left wrist with GSR nodes around their left ring and middle fingers and a PPG node on their left ear. A user wearing the equipment can be viewed in Figure 3. They were asked to confirm that they were comfortable and were able to see the VR environment clearly. If they responded with no, adjustments were made until they were comfortable. In the VR environment, they moved at their own pace, experiencing the environment until they were ready to start the tutorial. Once the tutorial started, the VR experience took them through each condition, asking for a SUDS rating after every condition, followed by a fifteen-second break. The participants were aware that they were free to quit at any stage of the experiment. Once they completed the VR section of the experiment, the experimenter removed all the equipment. They were then given the second questionnaire. After the experiment, they were given time to ask the experimenter questions.

### 2.7. Data Acquisition and Real-Time Processing

As detailed previously, it was decided that we will use Shimmer GSR+ for PPG and EDA and MyndPlay MyndBand for EEG. PPG has a training period of ten seconds, whereas the other measures can be used earlier. The EEG has a refresh rate of 512 Hz, where data from PPG and EDA stream every second.

The information collection in this experiment was carried out using the official APIs of the Shimmer and NeuroSky MyndPlay MyndBand devices. As such, it already includes some real-time processing, which is very useful for our real-time application. In the case of the PPG, the raw signal goes through real-time R-R peak detection, outputting the HR value. More information on the estimation can be found in the Shimmer validation paper [34].

Using the Shimmer API, a low-pass filter with a corner frequency of 5 Hz was applied to the PPG signal. Similarly, a high-pass filter was used to filter frequencies above 0.5 Hz. The PPG to heart rate calculations were carried out using the filtered signals. These can be viewed in Shimmer documentation [35].

In GSR signals, the noise is generally contained in high frequencies. Therefore, only a real-time low-pass filter was applied with a corner frequency of 5 Hz, as per Shimmer recommendations [35]. The low-pass filter used was Finite Impulse Response (FIR) Blackman-windowed-sinc.

All the preparation for the EEG signals was carried out using the NeuroSky software. The corresponding frequencies for each band are as follows: delta, 1–3 Hz; theta, 4–7 Hz; alpha1, 8–9 Hz; alpha2, 10–12 Hz; beta1, 13–17 Hz; beta2, 18-3 Hz; gamma1, 31–40 Hz; and gamma2, 41–50 Hz. An “e-sense” technology was used to compare each band every second. More information can be found in the Neurosky documentation [36].

As all the devices have different sampling rates, downsampling was required to synchronise them. The GSR signal was downsampled to 1Hz using the built-in methods from Shimmer API. In the case of EEG signals, the e-sense calculations from MyndPlay MyndBand ThinkGear software are outputted at 1 Hz. The heart rate calculations are also carried out in 1 Hz.

### 2.8. Statistical Analysis

We ran repeated measures ANOVA to compare the means of user skin conductivity, self-reported anxiety, and reaction time to detect the differences between three conditions: neutral, mild emotional, and severe emotional. We applied Greenhouse–Geisser correction to account for non-spherical data.

The skin conductivity levels averaged an increase of 31% between the neutral and severe emotional stages, and self-reported anxiety averaged an increase of four. Based on ANOVA results, there is a significant difference trend between conditions based on GSR (F = 20.182, *p* < 0.001) and self-reported anxiety (F = 3.877, *p* < 0.05). Bonferroni’s post hoc results revealed significant pairwise differences between stages for GSR (Table 1) but not for self-reported anxiety.

Reaction time decreased by 29 ms on average. There were no significant differences based on ANOVA results (F = 1.996, *p* > 0.05).

These results are in line with previous eStroop tests and VR Stroop tests [37,38,39,40,41]. A clear trend of increased arousal between conditions based on physiological information shows that our environment is suitable for machine learning (ML) classification.

### 2.9. Creating the Dataset

Data preparation was completed in real time, as detailed in the data acquisition subsection.

The values from before baseline detection and after the end of the experiment were manually removed using timestamps. All the separate .csv files for experiments were processed and combined into a single .csv file.

A simple method of train–test split was applied to compare different classification methods. The dataset was split into 80% training data and 20% testing data using the GroupShuffleSplit method.

The normalisation was applied for Artificial Neural Network (ANN), SVM, K-nearest Neighbour (KNN) and Convolutional Neural Network (CNN). The method used for normalisation is (value − mean)/std. It should be noted that the baseline-adjusted values are redundant in the case of normalised datasets, such as in the case of Neural Networks. Therefore, the baseline values were removed for these models, leaving only 15 features instead of 23.

Windowing was applied separately for CNN. All models except CNN took rolling means and standard deviations with window sizes of 3 s, 5 s, 10 s and 30 s and step sizes of 1 s, 1 s, 2 s and 10 s, respectively. Baseline-adjusted values were calculated by dividing each value by the mean of baseline recordings of the same metric.

For CNN, the dataset was iterated in window sizes of 3 s, 5 s, 10 s and 30 s and step sizes of 1 s, 1 s, 2 s and 10 s, respectively. The raw values of the window size of the rows were used as input. Each matrix of the window size × feature shape was labelled with the correct condition.

### 2.10. Feature Extraction

Firstly, relevant features were identified from previous literature. As our input is time-series data, we used all our metrics’ mean values and standard deviation over our sliding window. Since every person has different reactions and baseline values, we also included the mean values adjusted by the baseline. These were applied to the HR, GSR and EEG band wave values. The resulting features can be viewed in Table 2.

Adding minimum and maximum values was considered but decided against due to the short nature of the time windows and the results of direct experimentation with their inclusion.

Different feature extraction methods were applied to the CNN input. Instead of getting mean, baseline-adjusted mean and standard variation throughout the time window for our metrics, all metrics containing the row were used, forming matrices in the shape of time window × metrics. In this case, the extracted features were GSR, HR, delta, theta, low alpha, high alpha, low beta, high beta, low gamma and high gamma.

### 2.11. Feature Selection

An ANOVA test was used to determine the relationship between each feature and the condition to identify the most valuable features. The features with non-significant F values were discarded. These features were HR standard deviation, delta mean, delta mean-adjusted by baseline, delta standard deviation, high alpha mean adjusted by baseline, high alpha standard deviation and low gamma standard deviation. Features and their F values can be viewed in Table 2.

In the case of the CNN, the selected features were GSR, HR, theta, low alpha, low beta, high beta, low gamma and high gamma. Delta, high alpha and low beta were eliminated due to no relationship being found with the stage using ANOVA.

## 3. Results

Multiple models and window sizes were tested based on the properties data and prior literature.

For model comparison, 3 s time windows were used. A test–train split of 20–80 was applied, and the same validation and training groups were used to test each model. We used scikit-learn [42], TensorFlow Keras [43] and XGBoost [44] libraries for the models.

Our first tree model was a simple binary tree. We used parameters with twenty-four features and a maximum of twenty leaf nodes for baseline comparisons. When trained on our training set, it had 51% accuracy on our validation set. We next used a random decision forest. We used the default hyperparameters of the RandomForestClassifier method, with a maximum tree count of 100. We achieved 53% accuracy on the validation set. Finally, an extreme gradient boosted tree (XGBoost) classifier [44] was applied with default hyperparameters. We achieved 50% accuracy.

We used the K-Nearest Neighbours Classifier method with default hyperparameters and the number of neighbours set to 3. The training resulted in an accuracy of 62% on the validation set.

We used the SVM classifier with kernel set to poly and regularisation to 100. For the other hyperparameters, the defaults were used. Our training resulted in an accuracy of 63% on the validation set.

Considering our simple numerical dataset, we used a shallow neural network. We used the Keras Sequential model for training. The input layer has 64 neurons and uses activation relu. We have three dense layers with ten neurons each, and we use relU function for the activation. For the output layer, we used the softmax function. The selected hyperparameters were categorical_crossentropy for the loss function, Adam for optimiser and categorical_accuracy for metrics. We trained the dataset for 20 epochs with a batch size of 256 and used our validation set. There was slight overfitting, presumably due to the size of the training set. To overcome this problem, we added a dropout layer and performed the training with only 21 features, meaning a dropout of 0.1. This setup resulted in a 72% accuracy.

Due to the similarity of data formats, our CNN architecture closely followed our ANN architecture. Our first layer was a 0.2 dropout layer to combat overfitting. The second layer is a one-dimensional (temporal) convolutional (Conv1D) layer with 64 neurons, two kernels, and an input size of 5, 6. Five for our input shape represents the rows of data, in this case, 5 s of data. The six represents the number of features. We include six features in the training because we use a 0.2 dropout. We have our first dense layer with ten neurons and the relU activation function. This is followed by a pooling layer (MaxPooling1D) to downsample the features with a default pool size of 2. It has three more dense layers with a neuron size of 10 and finished with the activation layer, using the softmax function. The next layer is a flatten layer. We trained the dataset for 20 epochs with a batch size of 256 and used our validation set. We achieved 73% accuracy.

### 3.1. Leave-One-Out Cross-Validation

Although we used a simplistic approach of a 20–80 train–test split to compare different models as a means of determining accuracy, this approach has its drawbacks. As the dataset is not very large, how the split is made can greatly affect the accuracy found. This technique is sufficient when comparing models but not necessarily a good indicator of the model’s accuracy.

To achieve results with less bias, all parts of the data must be equally represented. There are several methods to achieve this. One of them would be k-fold cross-validation. K-fold cross-validation works by selecting k% of the data as validation data, training the model on the rest, saving the accuracy and then repeating k times for each section of the data. The accuracies calculated by each iteration are then averaged to obtain the final accuracy. A typical application would be 10-fold cross-validation, which trains and evaluates a model 10 times with a 10–90 split.

Similarly, leave-one-out cross-validation (LOOCV) works by splitting one data point or group and using the rest to train the model. This approach is a lot less prone to biases and gives us more information by taking every case into account. However, this can take a very long time in the case of extensive datasets. Since we do not have a large dataset, we decided to use LOOCV.

**Figure 4:**
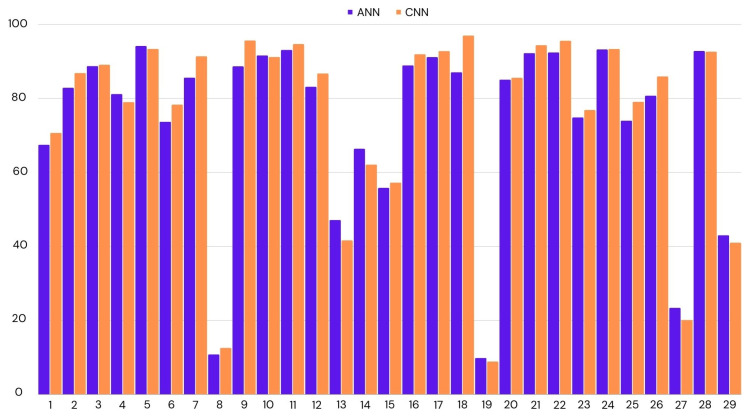
Classification accuracy for the each participant using leave-one-out cross-validation. ANN: Artificial Neural Network; CNN: Convolutional Neural Network.

We have applied LOOCV on both ANN and CNN, as they had similar accuracies. In the case of CNN, we achieved 75% accuracy across all participants, while this was the mean of all the accuracies, there were many outliers. If we look at the median score to represent the accuracy for most participants, it was 87%. The lowest accuracy was 9%, and the highest was 97%. We had similar results for ANN. The mean accuracy was 74%, with the highest accuracy being 94% and the lowest 10%. CNN performed better by a small margin. Figure 4 shows the distribution of accuracies per test participant.

**Figure 5:**
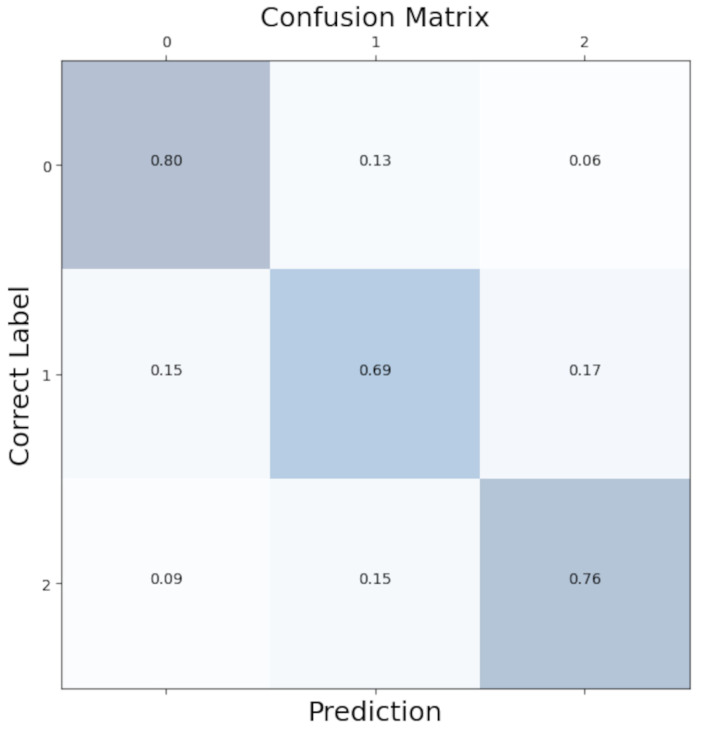
Confusion matrix for the Convolutional Neural Network. 0: no anxiety; 1: mild anxiety; 2: severe anxiety.

To better see the distribution of class predictions in our model, we aggregated confusion matrices from each LOOCV iteration to create an overall confusion matrix. As shown in Figure 5, class predictions were evenly distributed with minimal bias.

### 3.2. Window Selection

Deciding the sliding window size is one of the most crucial steps of time-series classification. Increasing the window size may improve accuracy. However, getting a more time-precise output from the classification method is critical for real-time usability. Therefore, we used the smallest time window while maintaining accuracy. We have experimented with 3 s, 5 s, 10 s and 30 s time windows.

We will benchmark the results of the windows using our best-performing algorithm, the CNN. We used LOOCV for each window size. Table 3 shows the accuracies achieved for each window size. It can be seen that different window sizes performed similarly, giving us a reason to use smaller window sizes for more precision in real-time.

## 4. Discussion

The protocol followed in this paper goes through the steps of data acquisition, data preparation, feature extraction and anxiety classification for use in real-time VR applications. It has many similarities to methods used in the literature for anxiety classification using biosensors. The methodology follows past examples of using well-established cognitive tasks to label data. It uses established metrics for classification as features: HR, GSR, alpha, beta and gamma EEG bands.

Convolutional neural networks are a common classification approach for time-series data, and they have the advantage of reducing data preparation time by not needing rolling windows. Many examples in the literature use simple convolutional network structures to analyse biosensor data for outputs such as emotion recognition and stress recognition [45,46,47].

The current work, however, differs in a few accounts. Firstly, for the purpose of real-time use, we used smaller windows and different data-cleaning methods. For example, applying Blackman filters per second can make extracting meaningful data more challenging than applying such filters over a long period. An instrumental metric, HRV, was also deemed impractical due to our short window lengths.

Additionally, our work has to deal with additional noise and unreliable signals due to VR electrical interference and excessive arm movements. Although biosensors produce much more accurate results when stationary, allowing arm movements is crucial for controls in VR. Our dataset was collected in a VR state-of-the-art emotional Stroop task environment to (1) guarantee that we are measuring mental anxiety and not task load and (2) that the data we collect will be similar, therefore applicable to our use case of VR therapy, and (3) while it is applicable to our scenario, it will also apply to different types of therapy by being a generalisable task.

We evaluate our model based on classification accuracy. Based on the leave-one-out cross-validation results, the model successfully predicts the correct label 75% of the time. However, it must be noted that based on the LOOCV results, there can be vast variations based on the specific participant, and while the median score was higher than 90%, the accuracy suffered with levels of around only 10% for two participants.

Our model correctly predicted the “no anxiety” condition 80% of the time and the “high anxiety” condition 76% of the time. Where the model has difficulties was the “mild anxiety” condition. In total, 15% of the mild conditions were incorrectly labelled as no anxiety, and 17% were incorrectly labelled as high anxiety. A total of 69% of the mild conditions were correctly predicted. This reflects the difficulty of clear-cut separations of fluid emotional anxiety levels.

The outliers in the model were examined for any possible explanations. Looking through the demographic information of these two participants, only one participant was an outlier in a category. They were one of the two people diagnosed with dyslexia in the dataset. Their dyslexia could be why they are an outlier; however, there is insufficient information to draw any conclusions. It must be noted that this method of anxiety classification might not be ideal for participants with dyslexia.

It must be noted that prior to machine learning analysis, it was planned that the neutral level of eStroop would be used for baseline analysis in practical applications. However, using real-time analysis simulations seems implausible due to not representing a wide range of anxiety. The dataset’s standard deviation and mean must be known for normalisation reasons. However, the mean is a lot lower in the neutral condition. Therefore, the normalisation and analysis are only accurate with the administration of the complete eStroop task.

### 4.1. Comparison to Similar Work

The literature that focuses on real-time anxiety detection for VR is limited due to complications with real-time signal processing as well as the limited research on VR. Some studies employed longer time windows, making it unsuitable for real-time predictions [48,49,50]. Many studies with shorter time windows have limited participant numbers or are preliminary studies [18,19,51,52]. Generalisation can be challenging in such studies. A summary of the comparison of our results with previous studies can be viewed in Table 4.

It can be seen that previous studies with three output levels and short time windows (3–30 s) range between 67% and 96% accuracy. The closest condition to our study was one conducted by Wu et al. [55], using the Stroop Colour–Word Task (SCWT) for stress inducement and classifying between three output levels. They also had 19 participants, closer to our study than the others. They achieved 84% accuracy using an SVM. However, it must be noted that in the case of traditional SCWT, it is difficult to differentiate mental load from emotional anxiety.

It must be noted that all of the studies in the literature use different methods for ground truth and labelling anxiety, which makes direct comparisons challenging. Our study differs from previous studies because we introduce a new way of measuring ground truth, which makes it implausible to do a direct comparison. Our study also avoids using computationally heavy processing or processing that requires full datasets so that it can be applied in real-time.

### 4.2. Limitations

There are some limitations due to the representation of the dataset sample. The dataset includes 29 participants recruited from a large university campus, which may not accurately reflect the general population. Additionally, our method of separating anxiety based on emotional words might not reflect the full breadth of the anxiety spectrum, making it challenging to separate very high levels of anxiety from high-level anxiety.

Furthermore, this study only addresses methods for decreasing noise in real-time using filters and does not propose any physical methods to account for noise in signals. We also do not have noise cancellation for possible scalp tension contamination of the EEG signal in 8–12 Hz frequencies [56]. We ensure our methods are validated by using validated APIs and software by publishers. However, we do not contribute to data acquisition methods.

We use raw signals for GSR and convert PPG to HR in real time. We also extract band information from raw EEG signals in real time. For ideal results, developing an algorithm that can separate phasic and tonic skin conductivity signals within a five-second timeframe would be necessary. Similarly, developing an algorithm that can reliably measure HRV from the raw PPG signal in this short time would be optimal.

## 5. Conclusions

This study showcases our real-time anxiety classification system for seamless and objective feedback in VRET. We were able to achieve 75% accuracy between three output classes, showing that we can predict anxiety in real time with no access to signal processing tools that rely on complete datasets.

We also emphasised the real-time aspect of our algorithm by keeping the sliding windows small at only 5 s. This increases the reliability of our results by showing more recent information. Some algorithms may claim real-time results, but their data use time windows of around a minute.

It must be duly noted that our results show that it can be challenging to apply the classification to some users with the same accuracy, as evidenced by our LOOCV results. For some participants, the classification accuracy was as low as 9%. It is essential that factors that cause such outliers are investigated for the general applicability of these results.

We plan to integrate our model into the Unity environment for real-time classification. By doing so, we aim to accomplish seamless decision support for the therapist. We aim to create a generalisable system for anxiety detection. However, thus far, we have only tested the system in the eVRST system. Therefore, the first step after integrating into the VR framework will be to validate it within the exposure environment.

There is some evidence of anxiety classification within VRET providing optimal results. However, no research exists to see if such systems with seamless feedback improve therapy outcomes. It is crucial to conduct experiments with actual patients to see the effect of such decision-support frameworks.

## Figures and Tables

**Table 1:** Bonferroni post hoc results of ANOVA for measuring differences in Galvanic Skin Response level between neutral, mildly emotional and severely emotional conditions.

Measure	(I) Stage	(J) Stage	Mean Difference (I–J)	SE	Sig.
GSR	1	2	−0.579	0.154	0.002
		3	−1.000	0.214	<0.001
	2	1	0.579	0.154	0.002
		3	−0.421	0.074	<0.001
	3	1	1.000	0.214	<0.001
		3	−0.421	0.074	<0.001

**Table 2:** The extracted features and their ANOVA F-values.

Measure	Feature	F
GSR	GSR_mean	115.3 ***
	GSR_baseline	2379.1 ***
	GSR_SD	3.9 *
HR	HR_mean	15.2 ***
	HR_baseline	18.4 ***
	HR_SD	0.5
EEG	delta_mean	2.4
	delta_baseline	1.8
	delta_SD	1.2
	theta_mean	7.1 ***
	theta_baseline	4.0 *
	theta_SD	3.6 *
	lowalpha_mean	10.9 ***
	lowalpha_baseline	9.0 ***
	lowalpha_SD	4.5 *
	highalpha_mean	5.8 **
	highalpha_baseline	2.8
	highalpha_SD	1.1
	lowbeta_mean	6.2 ***
	lowbeta_baseline	4.7 **
	lowbeta_SD	3.1 *
	highbeta_mean	15.4 ***
	highbeta_baseline	17.3 ***
	highbeta_SD	7.2 ***
	lowgamma_mean	9.8 ***
	lowgamma_baseline	4.2 *
	lowgamma_SD	1.7
	highgamma_mean	7.8 ***
	highgamma_baseline	8.8 ***
	highgamma_SD	4.6 **

*** *p* < 0.001, ** *p* < 0.01, * *p* < 0.05.

**Table 3:** Time windows used and the corresponding classification accuracy.

Time Window	Accuracy
3 s	74.50%
5 s	74.82%
10 s	74.74%
30 s	76.47%

**Table 4:** Comparison to similar studies.

Ref.	Sample Size	Features	Ground Truth	Highest Accuracy Model	Number of Outputs	Accuracy
[48]	8	HRV (ECG)	VR Content	LSTM	2, 3, 4	90.5% (2-Level), 67.5% (3-Level), 58.8% (4-Level), 30 s
[51]	4	PPG, EDA, EEG	SUDS	ANN	11, 4, 2	78.3% (2-Level), 38.8% (4-Level), 26.5% (11-Level)
[19]	12	PPG, EDA, SKT	VR Content, STAI-Y1	K-ELM	3	96.3%
[52]	6	ECG, PPG	VR Content	LDA	3	79%
[53]	28	EEG, EMDR	SCWT	MLP	2	96.42%
[18]	7	EDA, PPG	SUDS	Regression	2, 3	94.3% (2-Level), 92.4% (3-level)
[54]	20	ECG, RESP	STAI-Y1	Neuro-fuzzy	4	83%
[14]	30	BVP, EDA, SKT	VR Content, SUDS	SVM	4	86.3%
[55]	19	EDA, RESP, ECG, EEG	SCWT	SVM	3	84%
Our Study	29	EDA, EEG, PPG	eVRST	CNN	3	75.38%

Note: ANN: Artificial Neural Network; BVP: Blood Volume Pressure; CNN: Convolutional Neural Network; ECG: Electrocardiogram; EDA: Electrodermal Activity; EEG: Electroencephalogram; EMDR: Eye Movement Desensitization and Reprocessing; EMG: Electromyogram; HRV: Heart rate variability; K-ELM: Kernel Extreme Learning Machine; LDA: Linear Discriminant Analysis; LSTM: Long Short-Term Memory; MLP: Multi-layer Perceptron; PPG: Photoplethysmogram; RESP: respiration; SKT: skin temperature; SCWT: Stroop Colour–Word Task; SUDS: Subjective Units of Distress Scale; STAI: State-Trait Anxiety Inventory; SVM: Support Vector Machine.

## Data Availability

Publicly available datasets were analyzed in this study. These data can be found here: https://osf.io/dm4jb (accessed on 16 January 2024).

## Supplementary Materials

The following supporting information can be downloaded at: https://www.mdpi.com/article/10.3390/bios14030131/s1, Table S1: Affective Words.

## Abbreviations

The following abbreviations are used in this manuscript:

AI	Artificial Intelligence
ANEW	Affective Norms for English Words
ANN	Artificial Neural Network
ANOVA	Analysis of Variance
API	Application Programming Interface
BVP	Blood Volume Pressure
CBT	Cognitive Behavioural Therapy
CNN	Convolutional Neural Network
ECG	Electrocardiogram
EDA	Electrodermal Activity
EEG	Electroencephalogram
EMDR	Eye Movement Desensitization and Reprocessing
EMG	Electromyogram
eStroop	Emotional Stroop
eVRST	Emotional Virtual Reality Stroop Task
FIR	Finite Impulse Response
GSR	Galvanic Skin Response
HR	Heart Rate
HRV	Heart Rate Variability
HMD	Head-Mounted Display
ID	Identification
K-ELM	Kernel-Based Extreme Learning Machine
KNN	K-Nearest Neighbors
LDA	Linear Discriminant Analysis
LSTM	Long Short Term Memory
LOOCV	Leave-One-Out Cross-Validation
ML	Machine Learning
MLP	Multi-Layer Perceptron
PPG	Photoplethysmogram
RESP	Respiration
SCWT	Stroop Colour–Word Task
SKT	Skin Temperature
SNRI	Serotonin–Norepinephrine Reuptake Inhibitors
SSRI	Selective Serotonin Reuptake Inhibitors
STAI-Y1	State-Trait Anxiety Inventory-Y1
SUDS	Subjective Units of Distress Scale
SVM	Support Vector Machine
VR	Virtual Reality
VRET	Virtual Reality Exposure Therapy
